# The IMAGE beamline at the KIT Light Source

**DOI:** 10.1107/S1600577525003777

**Published:** 2025-06-02

**Authors:** Angelica Cecilia, Rolf Simon, Elias Hamann, Marcus Zuber, Tomáš Faragó, Daniel Haenschke, Mathias Hurst, Thomas van de Kamp, Sondes Bauer, Rebecca Spiecker, Mateusz Czyzycki, Sergei Gasilov, Alexey Ershov, Jan-Thorsten Reszat, Tilo Baumbach

**Affiliations:** a Institute for Photon Science and Synchrotron Radiation (IPS) Karlsruhe Institute of Technology (KIT) Hermann-von-Helmholtz-Platz 1 76344Eggenstein-Leopoldshafen Germany; b Laboratory for Applications of Synchrotron Radiation (LAS) Karlsruhe Institute of Technology (KIT) Kaiserstraße 12 76131Karlsruhe Germany; c Helmholtz-Zentrum Hereon, Institute of Materials Physics, Max-Planck-Straße 1, 21502Geesthacht, Germany; Bhabha Atomic Research Centre, India

**Keywords:** X-ray imaging, tomography, laminography, *in situ*, *operando*

## Abstract

The superconducting wiggler beamline IMAGE at the KIT Light Source is dedicated to full-field hard X-ray imaging applications in materials and life sciences, with a focus on high-throughput computed tomography, laminography experiments and systematic *in situ* and *operando* studies. With two experimental hutches, IMAGE provides space for its dedicated permanent experimental stations UFO-II and LAMINO-II as well as for flexible custom setups. IMAGE allows parallel beam imaging experiments in monochromatic, pink or white beam mode, with spatial resolutions ranging from ∼1 µm to ∼30 µm and a large horizontal and vertical field of view of up to 44 mm × 8 mm, respectively. Photon energies between 8 keV and 40 keV are provided by a double-crystal or double-multilayer monochromator optics with energy resolutions of 0.01% and 1.5–2.7%, respectively. The filtered white beam mode provides high flux densities of up to 7.5 × 10^14^ photons s^−1^ mm^−2^ at the sample position and energy spectra extending up to about 120 keV.

## Abstract 2

The superconducting wiggler beamline IMAGE at the KIT Light Source, dedicated to full-field hard X-ray imaging applications in materials and life sciences, with a focus on high-throughput computed tomography, laminography experiments and systematic *in situ* and *operando* studies, is described.

## 1. Introduction

Since their emergence in the 1990s, synchrotron microtomography techniques have been widely used in various disciplines, including life sciences, materials research, archaeology, medicine and palaeontology (Bowen *et al.*, 1986 ▸; Snigirev *et al.*, 1995 ▸; Takeda *et al.*, 1995 ▸; Cloetens, Ludwig, Baruchel *et al.*, 1999 ▸; Baruchel *et al.*, 2006 ▸; Baruchel *et al.*, 2008 ▸; Tafforeau *et al.*, 2006 ▸). Several X-ray imaging contrast modes have been developed, including absorption- and propagation-based phase contrast (Cloetens *et al.*, 2001 ▸; Cloetens, Ludwig, Van Dyck *et al.*, 1999 ▸), diffraction contrast (Lang *et al.*, 1983 ▸; Tuomi, 2002 ▸; Coan *et al.*, 2008 ▸) and fluorescence contrast (Rust & Weigelt, 1998 ▸), with spatial resolutions reaching the submicrometre range (Rack *et al.*, 2008 ▸; Rack *et al.*, 2009 ▸; Banhart, 2008 ▸). Subsequently, the increasing demand for synchrotron imaging techniques has driven the realization of imaging beamlines at almost all synchrotron light sources to meet the diverse needs of scientific communities (Espeso *et al.*, 1998 ▸; Stampanoni *et al.*, 2006 ▸; Weitkamp *et al.*, 2017 ▸; Rau, 2017 ▸; Yoneyama *et al.*, 2021 ▸; Bonnin *et al.*, 2024 ▸). Hence, synchrotron radiation is today a valuable tool for a broad range of X-ray imaging and tomography applications benefiting from high photon flux density, high spatial coherence and parallel beam propagation (Withers *et al.*, 2021 ▸).

Here we present the current state of the IMAGE beamline at the Karlsruhe Institute of Technology (KIT) Light Source. IMAGE was previously used for commissioning and application of a dedicated endstation for Microscopy test and Quality Assurance (called MIQA). After the transfer of MIQA to our new beamline at PETRA III/IV at DESY, Hamburg, Germany, a major upgrade programme has significantly changed both the purpose and the instrumentation of the beamline. The beamline is now dedicated to providing versatile and performant equipment for full-field, micrometre-resolution hard X-ray imaging for materials and life science applications, with a dual focus on systematic *in situ* and *operando* imaging studies (radiography, tomography and laminography) along with high-throughput tomography.

The beamline has two experimental hutches (see Section 2): Experimental hutch 1 enables the development/realization of highly flexible setups of custom configurations, designed for specialized applications. Experimental hutch 2 houses dedicated permanent imaging stations. Here, the novel LAMINO-II station is applied to synchrotron computed lamino­graphy, *i.e.* three-dimensional (3D) imaging of flat, laterally extended objects. LAMINO-II focuses, on the one hand, on *in situ* or *operando* studies, *e.g.* under mechanical load, and, on the other hand, on hierarchical imaging studies by screening of large sample areas and zooming into specific regions of interest (ROIs) in 3D. In contrast, the UFO-I^1^ station is designed for high-throughput microtomography with automated sample exchange and will be replaced by the newly constructed UFO-II station, which is designed for AI-assisted serial microtomography, *e.g.* for larger comparative morphological studies or non-destructive testing of large sample series. UFO-II is currently under commissioning and will become operational in autumn 2025. Once it reaches routine operation, it will permanently replace UFO-I.

The beamline optics can optionally provide both monochromatic X-ray beams with photon energies between 8 keV and 40 keV [double-crystal monochromator (DCM) and double-multilayer monochromator (DMM)] as well as filtered white beam with energies up to about 120 keV, with a beam size at the sample position of up to 44 mm × 8 mm (H × V). These features allow tuning the bandwidth, flux density and field of view (FOV) to the needs of the experiment. In the following we refer to the DMM beam as the ‘pink beam’.

The following sections, Sections 2–5, outline the layout of the IMAGE beamline and the integrated experimental infrastructure. The beamline’s design and the incorporation of experimental tools are presented, providing insights into its operational capabilities.

In Section 6, illustrative application examples are introduced to demonstrate the experimental capabilities of the beamline, with main focus on *operando* tomography applied to vanadium redox batteries and high-throughput tomography applied to biological samples. These examples serve not only to highlight the technical aspects of the beamline but also to emphasize its practical utility for scientific applications. Through a detailed examination of its design and applications, IMAGE emerges as a versatile and robust tool for X-ray imaging research, promising valuable contributions to various scientific endeavours.

## 2. Beamline layout, beamline source and optics

**Figure 1:**
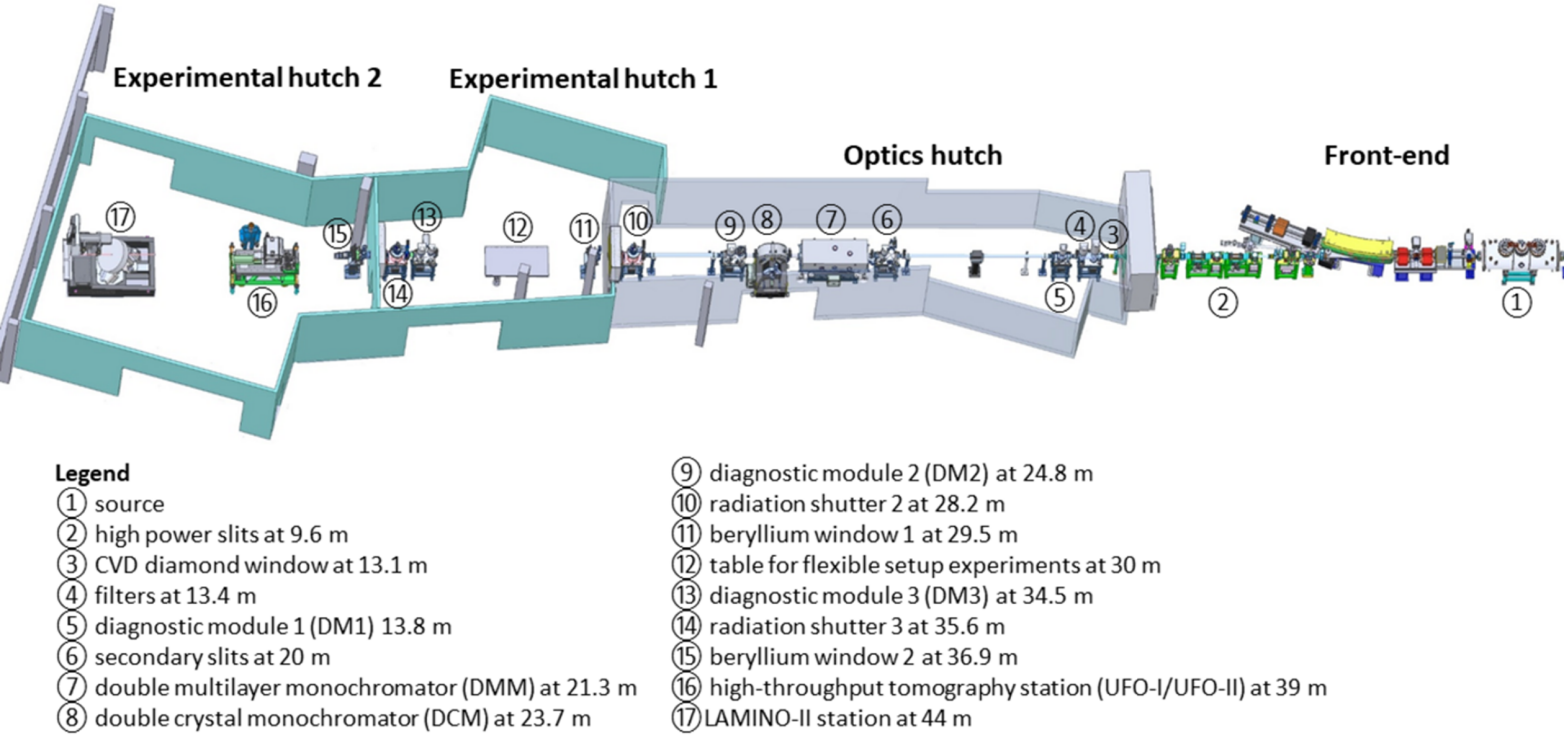
Schematic of the IMAGE beamline from the source to the experimental hutches.

The IMAGE layout illustrated in Fig. 1 ▸ shows the operational setup of the beamline, which comprises four sections:

(*a*) Front-end (1–2, Fig. 1 ▸), housing the wiggler source and the high power primary slits.

(*b*) Optics hutch (3–10), mainly hosting the monochromators and diagnostic modules.

(*c*) Experimental hutch 1 (11–14), providing work space for custom experiments at ∼30 m from the source.

(*d*) Experimental hutch 2 (15–17), dedicated to the permanent stations for tomography and lamino­graphy, located at 39 m and 44 m from the source, respectively.

### 2.1. Front-end

The radiation source of the beamline is a superconducting wiggler, which has been developed in the framework of the Compact Linear Collider (CLIC) collaboration among the European Organization for Nuclear Research (CERN), Budker Institute of Nuclear Physics (BINP) and KIT (Baumbach *et al.*, 2007 ▸; Bernhard *et al.*, 2013 ▸; Gerstl *et al.*, 2016 ▸). It can be operated in two different modes: (i) as a test facility, to investigate the impact of the wiggler on the beam dynamics in the storage ring, and (ii) as a light source, to produce the X-ray beam for the experiments at IMAGE. The main parameters of the CLIC wiggler are detailed in Table 1 ▸.

**Figure 2:**
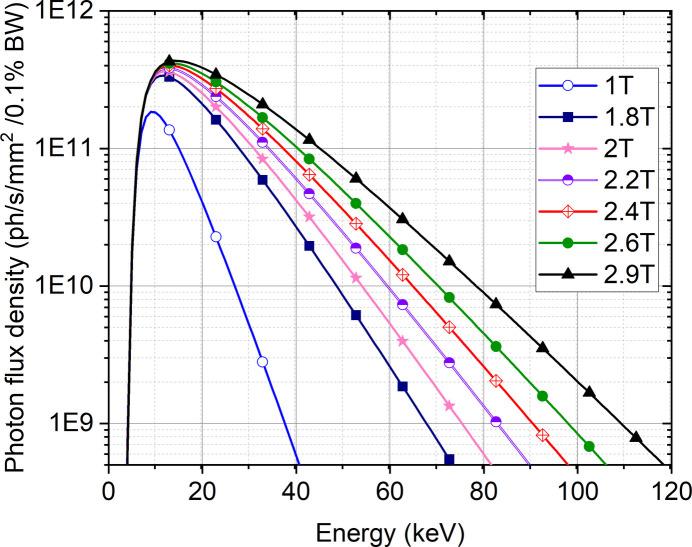
Energy spectrum of the wiggler for different magnetic field strengths. The spectra are calculated by using *SPECTRA 10.1* (Tanaka & Kitamura, 2001 ▸), for a distance of 39 m from the source and assuming a ring current of 100 mA.

By tuning the current in the superconducting coils, the magnetic field *B* of the wiggler can be varied between 1 T and 2.9 T, which allows adjustment of the critical energy *E*_c_ associated with the hardness of the source spectra (Peatman, 1997 ▸; Zhao & Fan, 2018 ▸). Fig. 2 ▸ shows the simulated flux density spectra at 39 m from the source, corresponding to magnetic fields between 1 T and 2.9 T. At the lowest magnetic field of 1 T, the critical energy is *E*_c_ = 4.08 keV and the energy spectrum extends with reasonable flux up to ∼40 keV. At the highest magnetic field of 2.9 T (*E*_c_ = 11.84 keV), the spectrum becomes considerably harder and extends up to 120 keV.

Furthermore, the change of the wiggler magnetic field also affects the deflection parameter and total power delivered from the source. In particular, the deflection parameter *K* (which measures the strength of the magnetic field of the wiggler in the midplane of the source) is linearly proportional to *B* via a proportionality constant equal to 4.76 T^−1^. The total power emitted by the CLIC wiggler follows a parabolic trend in relation to *B* (Peatman, 1997 ▸) with a constant coefficient of 0.67 kW T^−2^, and reaches a maximum value of 5.66 kW at the highest nominal magnetic field and with a ring current of 100 mA.

The lateral dimension of the wiggler source (1.254 mm × 0.065 mm, H × V @ 2.9 T) defines the transversal coherence length of the X-ray photons and is equal to 1 µm in the horizontal direction and to 18 µm in the vertical direction, assuming an energy of 20 keV and a distance from the source of 30 m. Due to the associated source blurring within the detected images, the horizontal dimension of the source size (*s*) determines the maximum propagation distance for phase-contrast imaging, equal to *D* = *PD*_0_/*s*, where *P* is the effective pixel size of the detector used and *D*_0_ is the sample distance from the source. Thus, typical maximum propagation distances range from a few centimetres up to several tens of centimetres for spatial resolutions between 1 µm and 30 µm, which match the available indirect detectors used at the beamline (see Table 5). When larger area detectors featuring larger pixels are employed, the propagation distance can be extended beyond 1 m. For applications requiring a longer transversal coherence length, the horizontal effective source size can be reduced by closing the primary slits, as proposed by Sun *et al.* (2022 ▸), or increasing the propagation distance. The closest relative position between the sample and the detector required for absorption imaging is equal to a few millimetres.

### 2.2. Optics hutch

In the following paragraphs the elements of the beamline X-ray optics are listed with increasing distance from the source. A 0.5 mm-thick chemical vapour deposition (CVD) diamond window (at 13.1 m from the source) separates the optical vacuum section from the vacuum front-end section. It can withstand a thermal load of 530 W distributed over a surface of 15 mm × 3 mm (H × V) and a maximum heat load density of 10.3 W mm^−2^, corresponding to the nominal CLIC wiggler magnetic field of 2.9 T and to a ring current of 200 mA. Given a decreasing magnetic field, the heat load on the window does not reduce like the total power emitted by the wiggler, because changes in the spectral and spatial distribution increase the probability of photon absorption in the window. This leads to a maximum heat load between 1 T and 1.5 T, which would require additional consideration in case the beamline is operated with more than 180 mA ring current at those magnetic fields.

**Figure 3:**
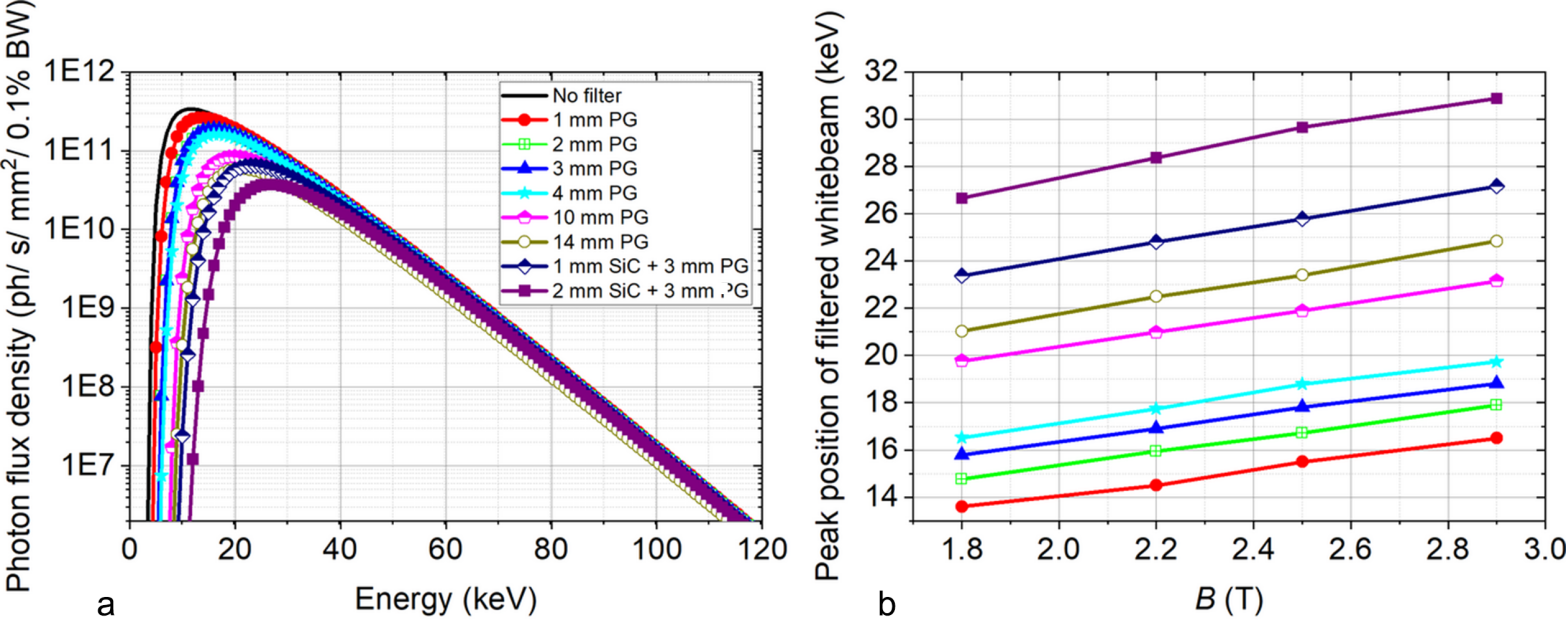
Energy spectra calculated for filtered white beam mode using different combinations of PG and SiC filters for a magnetic field of 1.8 T at 39 m from the source (*a*) and the corresponding energy peak positions using the same filter combinations for different magnetic field strengths (*b*). The spectra are calculated using *SPECTRA 10.1* (Tanaka & Kitamura, 2001 ▸). The colour code used for the different filter combinations on the left and right panels is the same.

Two filter units (at 13.4 m) with thin sheets of pyrolytic graphite (PG) and silicon carbide (SiC) ranging in thickness from 1–4 mm and 1–2 mm, respectively, provide thermal protection for subsequent optical components. In white beam mode, the magnetic field of the CLIC wiggler allows tuning the high-energy cut-off (Fig. 2 ▸), and the filters can be used and combined to shape the energy spectrum by eliminating undesired low photon energy components. As an example, Fig. 3 ▸(*a*) shows the calculated photon flux density at 39 m from the source with a magnetic field of 1.8 T by using different combinations of filters. By regulating the wiggler magnetic field for each filter arrangement, the peak position of the flux density spectrum can be adjusted between 13 keV and 31 keV, as shown in Fig. 3 ▸(*b*), tailoring the X-ray beam properties to the necessity of the experiment. If required, a set of additional, external filters (Si, Al) are available.

A set of water-cooled white beam slits positioned a distance of 20 m from the source allow the position and beam size to be defined before it enters the monochromators.

### 2.3. Double-multilayer monochromator

A DMM from XDS Oxford (https://xds-oxford.com/) is positioned at 21.3 m from the source and provides the polychromatic pink beam mode with moderate energy bandwidth. Full tunability over an energy range from 8 keV to 40 keV has been achieved by depositing two different multilayers [Pd/B_4_C and W/B_4_C, provided from AXO (https://www.axo-dresden.de/en/)] side by side on the same silicon (Si) substrate, 400 mm long, 100 mm wide and 40 mm thick. The layers, which are 390 mm long and 35 mm wide, are 10 mm apart.

**Figure 4:**
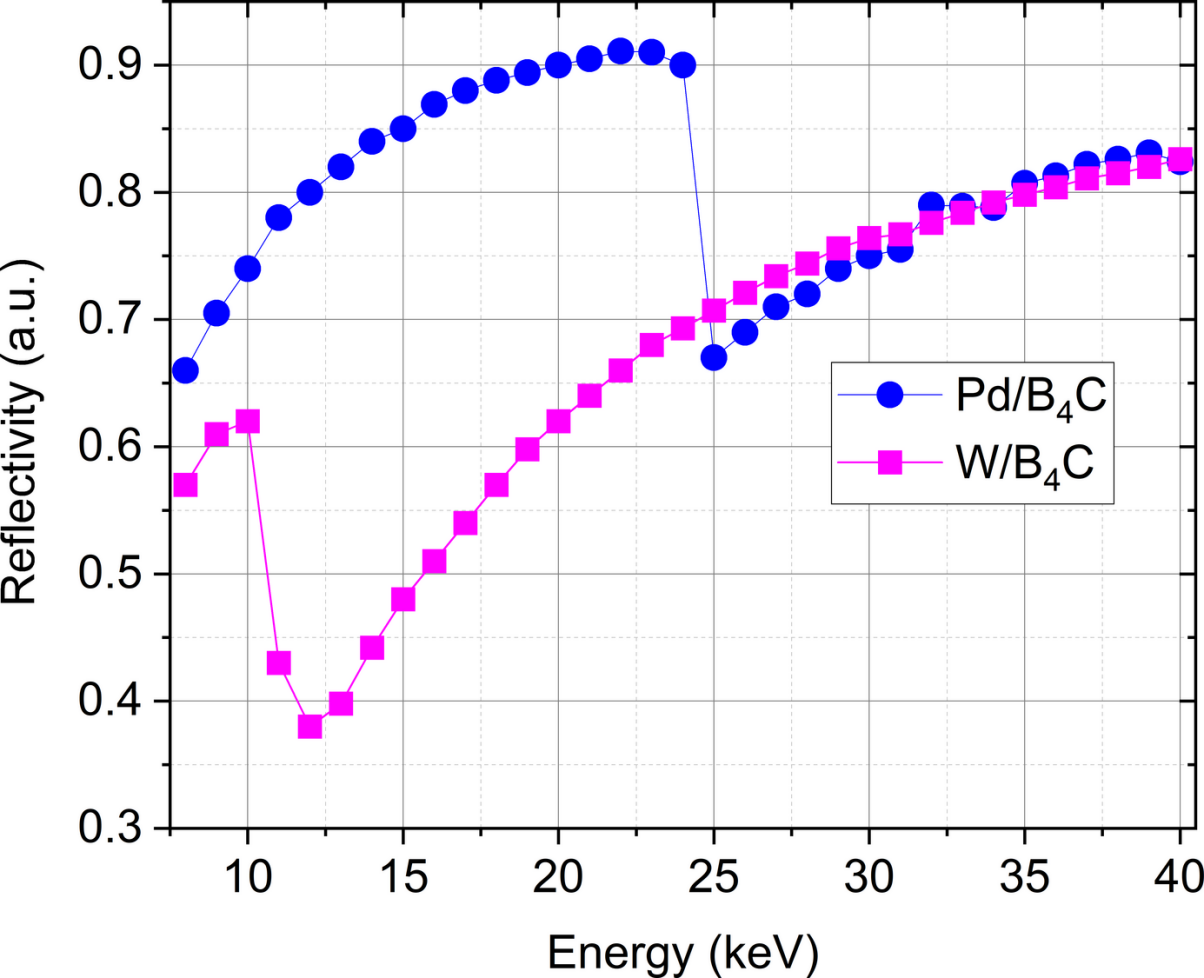
Reflectivity of multilayer stripes in the energy range 8–40 keV [calculations performed with the module *IMD* of *XOP* (Dejus & Sanchez del Rio, 1996 ▸)].

The multilayer structure used in the energy range between 8 keV and 20 keV consists of 70 bilayers of Pd/B_4_C, with a period thickness of 3.5 nm, and is optimized for 10 keV (peak reflectance of 75.3%) with 2.7% energy bandwidth. The second multilayer structure, comprising 110 bilayers of W/B_4_C with a period thickness of 2.2 nm, is optimized for 40 keV (peak reflectance of 82.5%) and provides X-ray photons in the energy range 20–40 keV with 1.5% energy bandwidth. For each stripe, a thickness ratio of 0.5 has been chosen to minimize the higher second diffraction order (Koyama *et al.*, 2022 ▸). The reflectivity of the multilayers in the working energy range of the DMM is shown in Fig. 4 ▸. The calculation, performed using the module *IMD* of *XOP* (Dejus & Sanchez del Rio, 1996 ▸), assumes a layer diffuseness of 6 Å. By combining the angle of incidence range of the two multilayers, *i.e.* 0.53–1.32° for Pd/B_4_C and 0.41–0.83° for W/B_4_C with their respective DMM vertical beam offsets relative to the white beam (14.5 mm and 12.5 mm), it is possible to cover the energy range 8–20 keV with the Pd/B_4_C and 20–40 keV with the W/B_4_C, all within a relative longitudinal shift of the crystals between ∼300 mm and 900 m.

**Figure 5:**
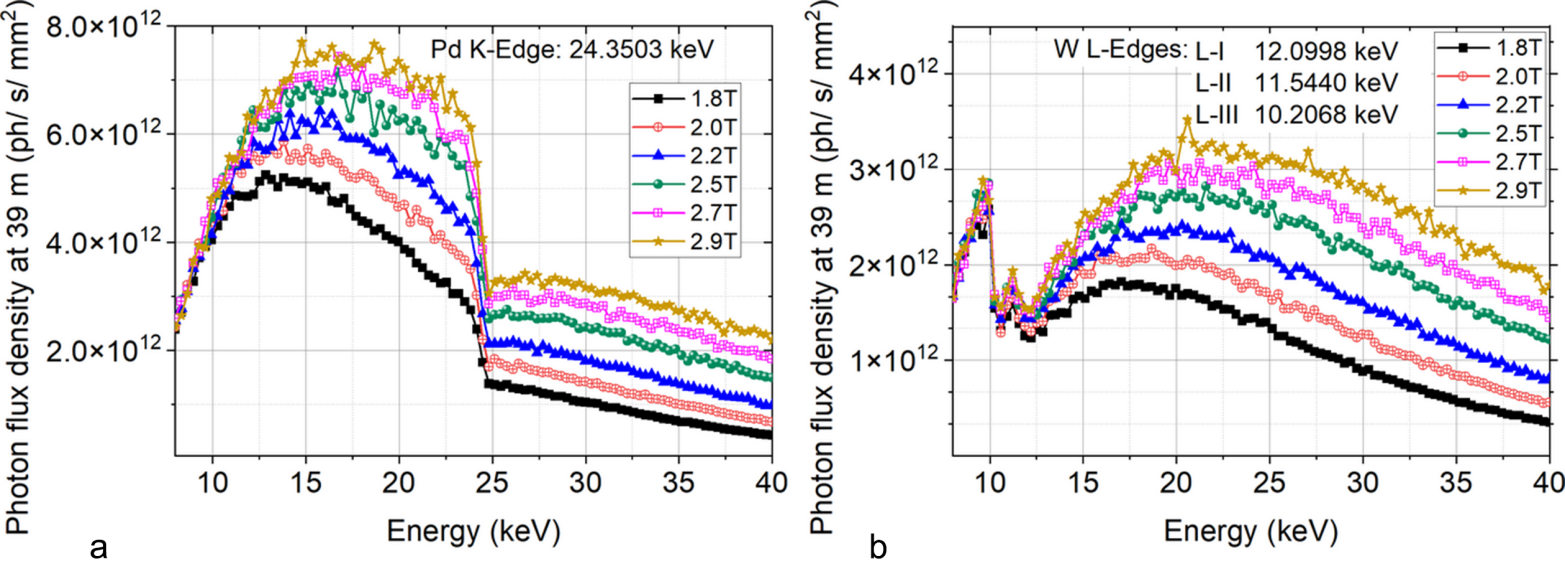
DMM photon flux density calculated at 39 m from the source (ring current = 100 mA, no filter). (*a*) Diffracted spectrum from Pd/B_4_C; (*b*) beam diffracted from the W/B_4_C multilayer (calculations performed with *XTRACE*). The simulations refer to the on-axis performance of the DMM (for off-axis performance, see the supporting information).

DMM flux density spectra calculated at the sample position with the beamline/X-ray optics simulation software *XTRACE* (Bauer *et al.* 2007 ▸) are shown in Fig. 5 ▸ for selected values of the wiggler’s magnetic field (at the ring current of 100 mA). Each spectrum clearly distinguishes the Pd *K*, W *L*-I, *L*-II and *L*-III edges, therefore emphasizing the selection of heavy elements for distinct energy ranges. The tungsten-based multilayer operates undistorted around the W *L*-edges at energies above 20 keV, while the palladium-based multilayer proves effective below the Pd *K*-edge at 24 keV.

The DMM has been specifically engineered to accommodate a maximum white beam size of 31 mm × 5.5 mm (H × V) and sustain an impinging heat load of 1247 W at the wiggler nominal magnetic field of 2.9 T. To manage the thermal load, the multilayer substrate mirrors are actively cooled down with a liquid nitro­gen cryocooler. Under an impinging heat load of 1247 W, the cooling system maintains the temperature of the first crystal at around 120 K close to the zero-point-crossing of the coefficient of thermal expansion and the temperature of the second crystal below ∼100 K (Rodriguez *et al.*, 2018 ▸). Such temperature control minimizes distortions caused by thermal gradients, given the small ratio between the thermal expansion coefficient and the thermal conductivity of the silicon substrate material.

While the DMM produces a pink beam suitable for many imaging applications, the intensity distribution of the outgoing beam is usually distorted by stripe patterns caused by phase shifts induced by even the smallest imperfections of the multilayer mirrors (Rack *et al.*, 2010 ▸). These patterns can challenge conventional flat-field correction methods when they exhibit time-dependent variations attributed to the overlapping effects of various origin such as ring orbit instability, heat load and temperature variations induced by the cooling system. In severe cases, advanced flat-field correction methods that account for time-dependent variations of the DMM stripes are required (Van Nieuwenhove *et al.*, 2015 ▸). For this reason the analysis of DMM pattern evolution over a long time period was an important part of beamline characterization. It was conducted at IMAGE over one hour and six hours of monitoring, respectively, and demonstrated vertical shifts of the beam of less than 1.3 µm within the initial hour of monitoring and ∼6 µm after six hours of continuous observation, indicating a stable beam and opening options for an efficient flat-field correction.

### 2.4. Double-crystal monochromator

For energy and phase-sensitive X-ray imaging applications requiring higher energy resolution, IMAGE has a DCM (from XDS Oxford) in vertical Bragg reflection geometry. It is positioned at 23.7 m from the source and it is equipped with a Si(111) crystal pair with dimensions of 120 mm in length, 40 mm in width and 40 mm height. This configuration enables choosing monochromated X-ray photons between 8 keV and 40 keV with d*E*/*E* of the order of 10^−4^, maintaining a fixed exit and at a vertical beam shift of 25 mm compared with the white beam position. The DCM is designed to accept a white beam size of 32.8 mm × 5.7 mm (H × V) with an incident power of 1247 W. The silicon crystals are cryogenically cooled by a liquid-nitro­gen cryocooler in order to maintain the temperature of the first crystal at around 120 K and the temperature of the second crystal below ∼100 K. Finite element analysis (FEA) calculations performed by the manufacturer verify the effectiveness of the cooling system at a photon energy of 22.68 keV: the RMS error of the tangential slope is 14% of the Darwin width of the Si(111) crystal, thereby offering excellent transmission under the applied heat load.

**Figure 6:**
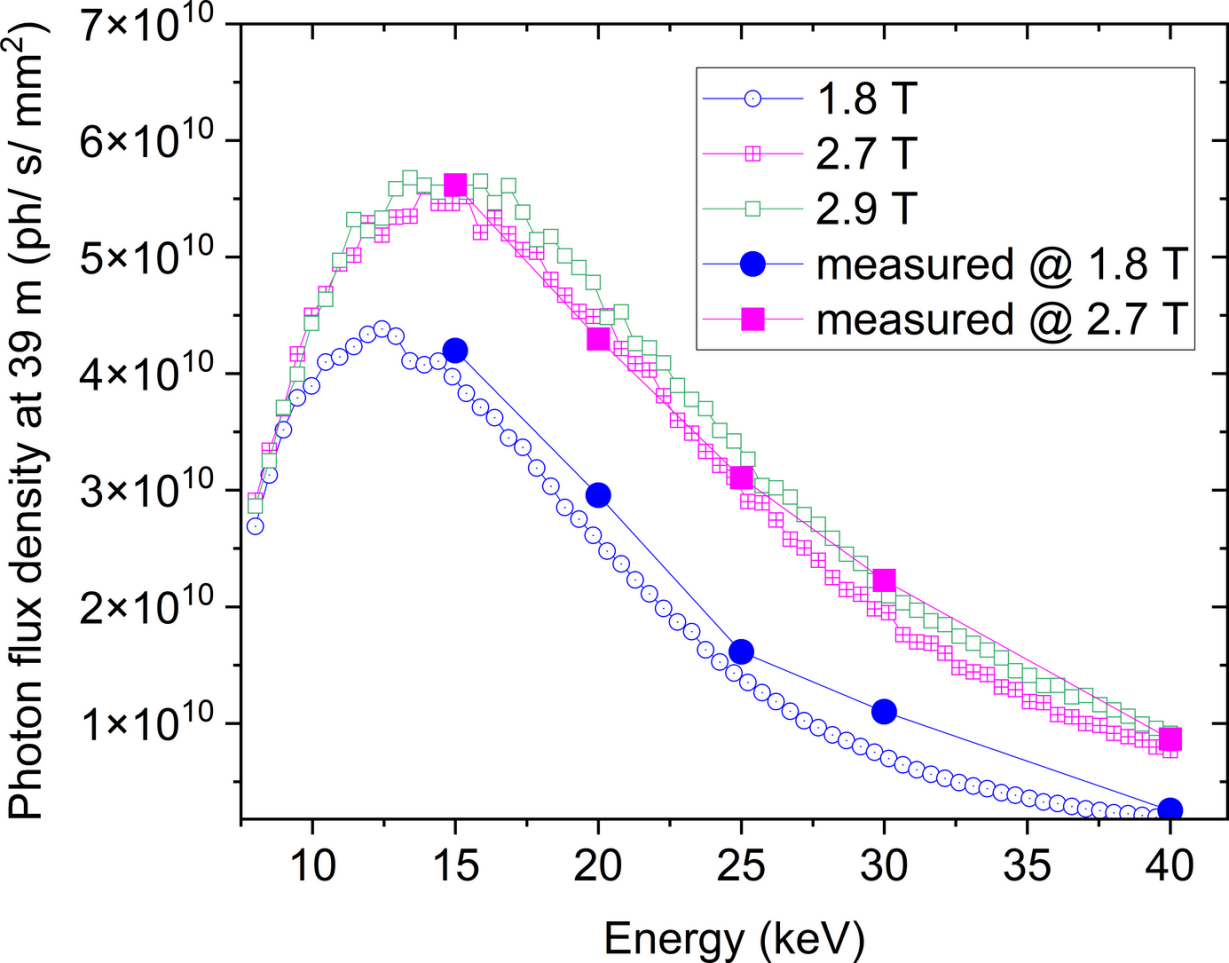
Calculated DCM photon flux densities at 39 m from the source for different magnetic fields of the wiggler (calculations performed with *XTRACE*). The solid circles/squares correspond to the flux density measurements performed using a Hamamatsu S3590-09 diode at 1.8 T and 2.7 T magnetic field, respectively. The simulations and experimental points refer to the on-axis performance of the DCM (for off-axis performance see the supporting information).

The DCM spectra calculated with *XTRACE* at 39 m from the source at magnetic fields of the wiggler of 1.8 T and 2.7 T are shown in Fig. 6 ▸ together with the photon flux densities measured with a Hamamatsu S3590-09 diode for representative energies. The experimental points match the *XTRACE* calculations (Bauer *et al.*, 2007 ▸) within a tolerance of less than 10%, validating the accuracy of the photon flux measurements.

### 2.5. Diagnostic modules

The IMAGE beamline integrates three diagnostic modules (DMs) for beam inspection: DM1, located in the Optics hutch at ∼13.6 m (and therefore used only for the white beam), DM2, positioned at 24.8 m from the source, and DM3, located in Experimental hutch 1 at 34.5 m from the source. These modules employ visible light fluorescence screens and X-ray beam intensity monitors for beam alignment, as well as for monochromator fine-tuning. A profile monitor consisting of two Si photodiodes (one for the DCM and DMM beams and the other one for the white beam) is located in DM2 to measure the 2D beam profiles at 24 m from the source, by scanning the diodes in the plane perpendicular to the beam direction. Details of the diagnostic modules components (sensors and materials) are available in Table 2 ▸, and a list of units installed in each diagnostic module is presented in Table 3 ▸.

## 3. Experimental hutches and experimental stations

IMAGE has two experimental hutches, indicated in Fig. 1 ▸ as Experimental hutch 1 and Experimental hutch 2. The beam enters each experimental hutch through a 300 µm-thick Be window (54 mm × 12 mm, H × V) installed on a motorized stage that allows the vertical position of the window to be adjusted to the three operational modes of the beamline (white beam, pink beam and monochromatic beam modes). Each beryllium window is protected against radiation and air contamination by keeping it in a helium atmosphere closed downstream with a 30 µm-thick kapton foil. The instrumentation in Experimental hutch 1 features high flexibility for custom experiments, *e.g.* with non-standard sample environments. The Experimental hutch 2 houses the permanent stations dedicated to *in situ* and *operando* lamino­graphic imaging and high-throughput tomography.

### 3.1. Experimental hutch 1

**Figure 7:**
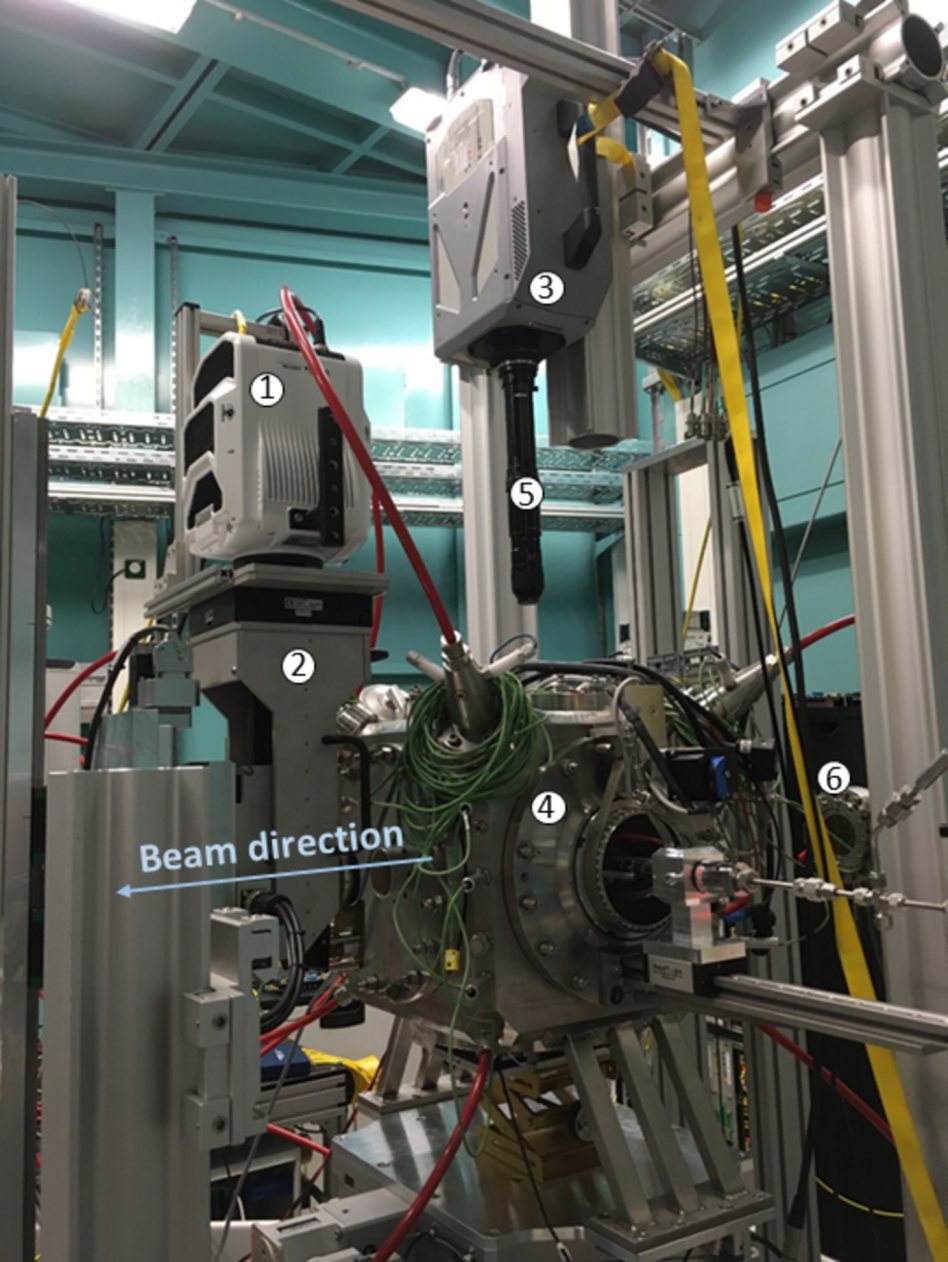
Custom set-up used in Experimental hutch 1 for *operando* tomography of injection sprays: (1) Phantom v2640 camera, (2) macroscope, (3) Photron Fastcam SA-Z camera, (4) injection chamber, (5) Navitar long-distance microscope objective, (6) beam exit.

Experimental hutch 1 is devoted to conducting ‘non-standard’ experiments that require the use of custom and/or large equipment such as test chambers and dedicated sample environments. To illustrate the flexibility, Fig. 7 ▸ details an example of such a custom configuration, which was used to accomplish the *operando* visualization of spray gasoline injections by combining X-rays and visible light imaging techniques. Further details about the used technique are available in the work of Bornschlegel *et al.* (2021 ▸).

The experimental set-up uses an X-ray indirect detector, consisting of a Phantom v2640 camera (https://www.phantomhighspeed.com/) coupled to a macroscope (3× magnification, effective pixel size of 4.33 µm) with a 200 µm-thick LuAG scintillator, used to visualize the injected spray in the vicinity of the nozzle area. The visible light detector, composed of a Photron Fastcam SA-Z (https://photron.com/contact-photron/) coupled to a Navitar long-distance microscope objective (https://www.navitar.com/), is used to acquire the visible light images of the spray, especially at increasing distances from the nozzle where the spray density is very low. The injection chamber has an adjustable internal pressure from 0.3 bar (absolute) to 14 bar (absolute), and it is continuously purged with air to flush out the injected fuel. The injector can be rotated up to 360°, ensuring the possibility to perform *operando* tomography.^2^

### 3.2. Experimental hutch 2

Experimental hutch 2 houses the main experimental stations of the beamline: the high-throughput tomography station UFO-I that will be replaced in the near future by the UFO-II table (currently in the commissioning phase), and the LAMINO-II station.

**Figure 8:**
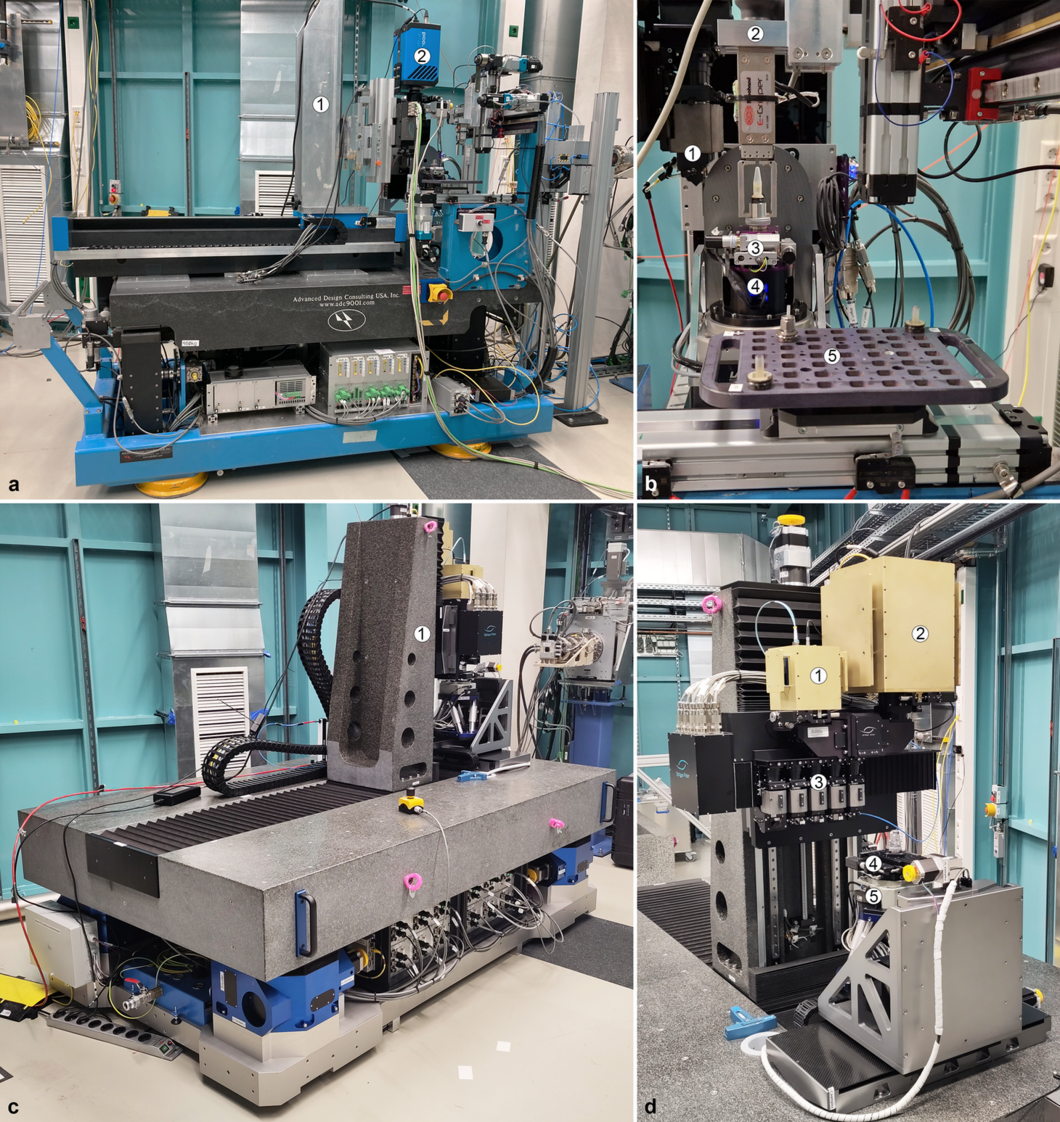
(*a*) UFO-I station for high throughput tomography in Experimental Hutch 2: (1) motorized detector tower, (2) pco.dimax camera. (*b*) Detail of the UFO-I sample exchanger with a tray for 49 samples: (1) white beam microscope, (2) sample changer gripper, (3) sample *x*–*y* linear stages, (4) rotary stage, (5) sample tray. (*c*) UFO-II during commissioning in Experimental Hutch 2: (1) detector tower. (*d*) Detail of the UFO-II quintuple magnification microscope and sample manipulator: (1) radiation shielded pco.edge5.5 camera, (2) radiation shielded Phantom v2640 camera, (3) visible light microscope with five different magnifications and camera branch selector, (4) sample *x*–*y* linear stage, (5) rotary stage.

The UFO-I station, positioned at 39 m from the source, provides high-throughput microtomography in absorption- and propagation-based phase contrast with a detector-to-sample distance up to 1.5 m (examples available in Section 6.2). The table axes (height, pitch and yaw) are motorized and its legs are equipped with air-bearing systems that allow moving the complete station (*i.e.* in and out of the beam path) on the floor of the experimental hutch [Fig. 8 ▸(*a*)]. UFO-I is supplied with a robotic system for an automated and quick sample exchange with up to 49 samples per tray [Fig. 8 ▸(*b*)].

For sample heights larger than the available FOV, the instrumentation (and processing pipelines) allows automatizing the recording of tomography scans at different vertical positions of the sample or to employ helical/spiral computed tomography measurements with combined rotary and vertical sample movements. The indirect detector, composed of two branches (one for high spatial resolution and the other one for low spatial resolution applications), is installed on a motorized stage to ensure its horizontal and vertical adjustment in the beam. Further details about the indirect detectors in use at IMAGE are available in Table 5.

The UFO-II station has been developed and assembled within a KIT in-house project. Compared with UFO-I, it offers higher mechanical stability, extended and more precise degrees of freedom for sample and detector positioning, as well as enhanced functionalities for high-throughput and hierarchical imaging. Besides providing all necessary motorized degrees of freedom, the station is equipped with a unique indirect X-ray detection system allowing automatic and quick switching between five different magnifications in the range 1× to 20× and between two different camera systems [see Figs. 8 ▸(*c*) and 8(*d*)]. A comparison of the main characteristics of UFO-I and UFO-II is given in Table 4 ▸. A typical complete scan cycle for one tomogram on the UFO-II station amounts to ∼30–60 s in white beam mode for a spatial resolution of ∼1 µm. In order to achieve fully automated scan sequences of large sample series without any user interaction, UFO-II will be equipped with a tray handling a sample exchanger system with capabilities of storing up to 2000 specimens with dimensions up to 2 cm × 2 cm × 5 cm, each having a unique ID (*e.g.* QR code or RFID chip) for data processing, storage and QA. Automated reconstruction pipelines based on the image framework *tofu* (Faragó *et al.*, 2022 ▸) are available. They find the 3D reconstruction parameters, suppress ring artefacts, perform phase retrieval, 3D reconstruction in both absorption and phase contrast modes and they blend the phase and absorption images. Processing one sample takes about 30 min including all intermediate steps and post-processing. Data transfer is done via hard-drive or access to a large data storage facility and raw data are archived in long-term storage.

**Figure 9:**
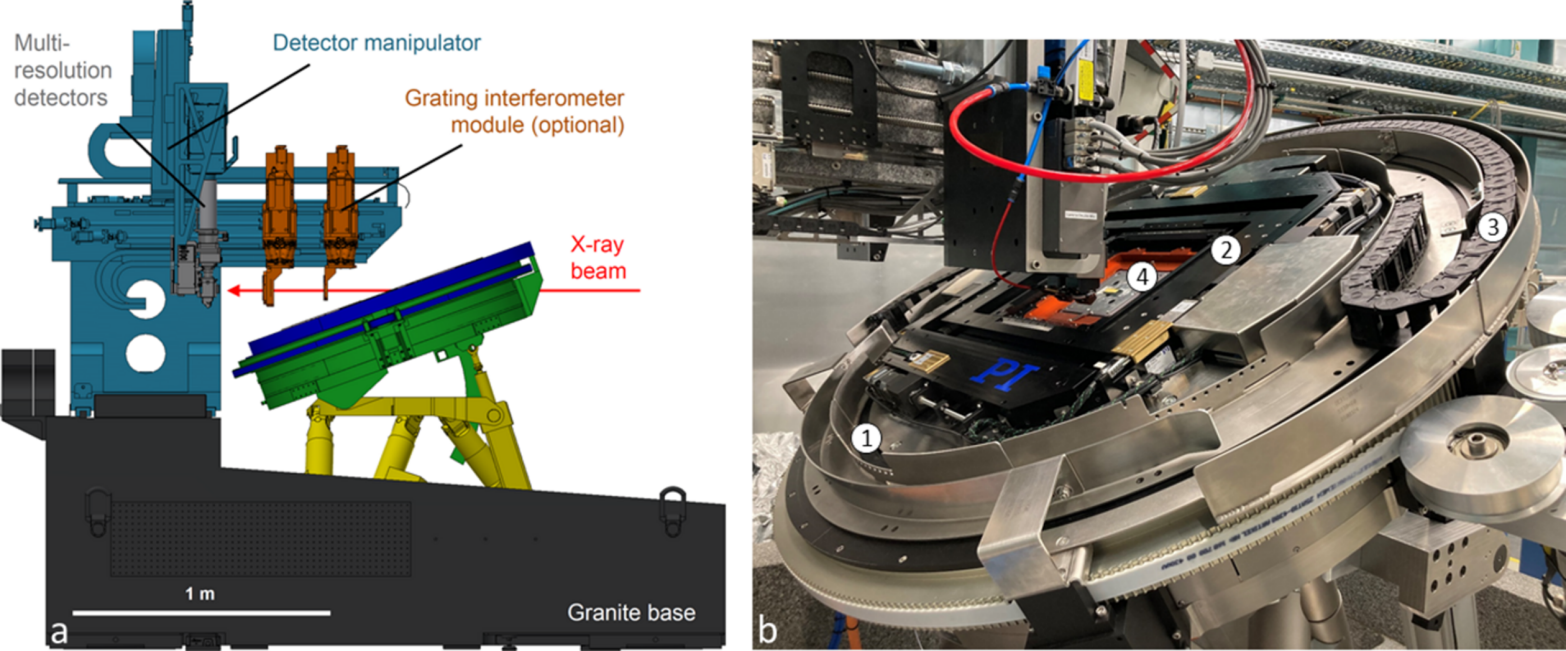
(*a*) Schematic side view of the LAMINO-II station highlighting the positioning modules for sample, detector and optical elements. (*b*) Photograph of the sample manipulator comprising a large air-bearing rotation axis (1) with open aperture in its centre, an *x*–*y* cross-table (2), as well as a cable drag (3) and a sample altogether tilted to an angle typical for lamino­graphic measurements of a sample (4).

The LAMINO-II station, positioned at 44 m from the source, has been designed for 3D X-ray computed synchrotron lamino­graphy of flat and laterally extended objects, whose dimensions significantly exceed the FOV of the detector (Helfen *et al.*, 2005 ▸; Helfen *et al.*, 2009 ▸; Helfen *et al.*, 2011 ▸). A schematic side view of the station highlighting the positioning modules for sample, detector and optional optical elements (such as, for example, a grating interferometer) is shown in Fig. 9 ▸ as well as a picture of the sample translations on top of the large rotation axis tilted to an angle typical for lamino­graphic measurements of a sample.

With the design of the LAMINO-II station, we aim for systematic *in situ* and *operando* studies down to micrometric 3D spatial resolution, in particular for materials testing, and hierarchical imaging, which can be done by screening large sample areas followed by zooming in on features in selected regions of interest. The station is equipped with an indirect X-ray detector system with two objective branches for motorized adjustment of effective pixel size/FOV (see Table 6 for details). If required, an optical microscope is available on the second detector arm for correlative imaging experiments.

**Figure 10:**
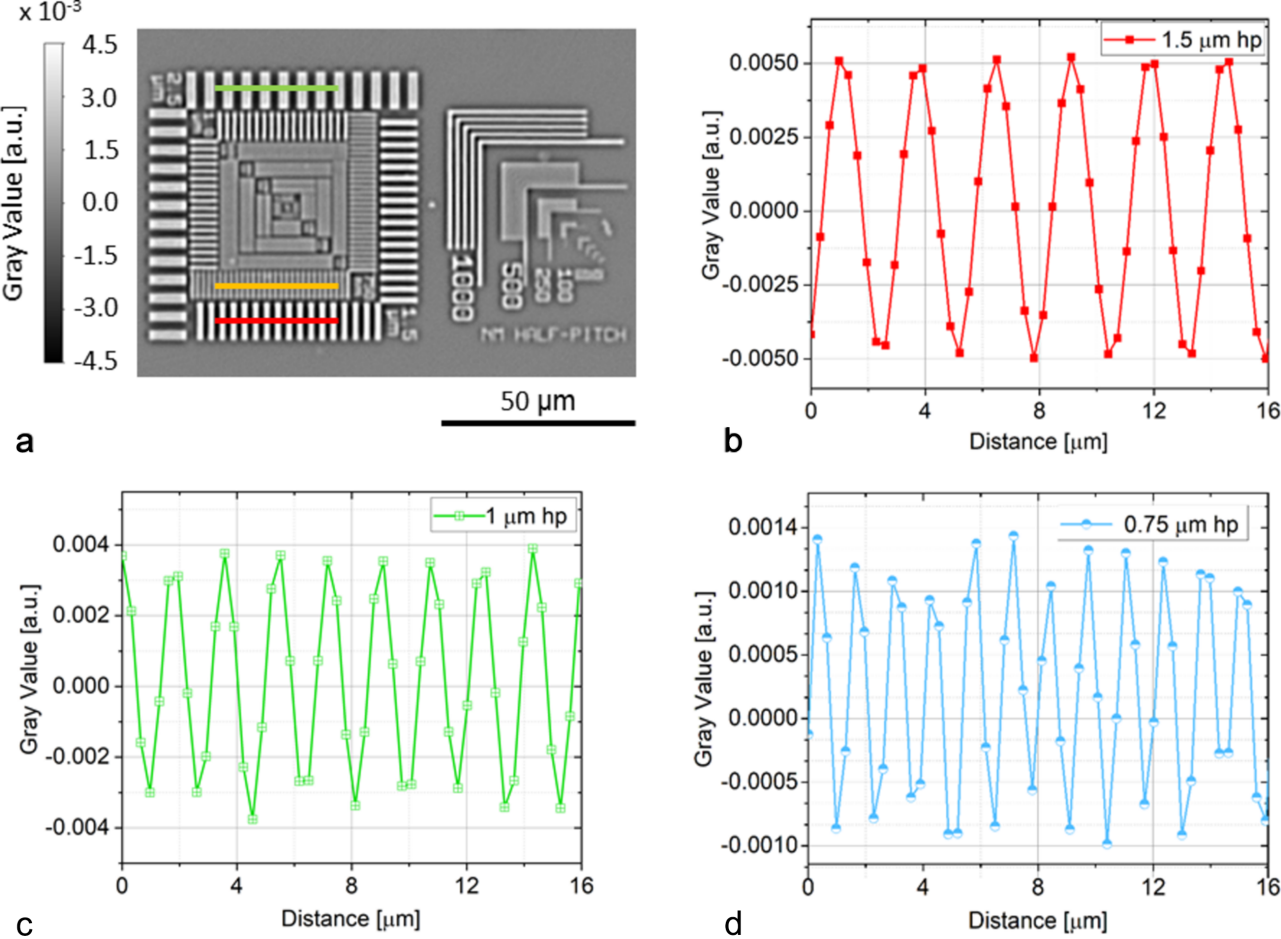
(*a*) Lateral slice through a 3D lamino­graphic reconstruction (0.36 µm effective pixel size) of a resolution test pattern (model ANT HXRCAL 25 nm, Edmonton, Canada), measured by LAMINO-II at IMAGE with a 45° tilt angle and with filtered whitebeam (*B* = 1.8 T, 3 mm PG and 3 mm SiC filters, 3000 projections for a total measurement time of 10 minutes). The sample-to-detector distance of ∼40 mm in combination with sufficiently closed primary slits of the beamline leads to features typical for beam propagation in the edge-enhancement regime (Cloetens *et al.*, 2001 ▸). (*b*)–(*d*) Grey value line profiles along the respective 1.5 µm, 1 µm, and 0.75 µm half pitch pattern. While from 1.5 µm to 1 µm the contrast decreases only slightly, it is strongly reduced for 0.75 µm, from which we estimate the achieved 3D spatial resolution to 1–2 µm.

The station was designed and constructed by PI miCos GmbH (https://www.physikinstrumente.de/de/ueber-pi/die-pi-gruppe/pi-micos/impressum) in collaboration with KIT. Special care was taken to allow imaging at 1–2 µm 3D spatial resolution (see Fig. 10 ▸) with up to 45° tilt even for relatively heavy (up to 4 kg) and considerably laterally extended (up to 250 mm × 250 mm) samples, which is necessary, for example, to investigate the 3D microstructural behaviour of metal sheets during *in situ* mechanical loading (Hurst *et al.*, 2023 ▸; Buljac *et al.*, 2023 ▸; Kong *et al.*, 2022 ▸). The lamino­graphic tilt angle can be adjusted from 20° to 45° and the air-bearing rotary stage with a large central aperture can rotate at a speed of 90° s^−1^ with a sphere of confusion of 0.7 µm, all the while avoiding obstruction of the beam path by anything but the sample. To avoid degrading the rotational precision because of the cables for power or media supply of sample environments, a dedicated cable drag is available, moving synchronously with the rotation axis in a leader–follower configuration [see Fig. 9 ▸(*b*)]. Finally, samples can be positioned and successively rasterized by the lateral *x*–*y* sample translation stage (75 mm × 75 mm) on top of the rotary axis, enabling fast and automated screening of large sample regions or following moving ROIs during *in situ* or *operando* measurements.

## 4. Control system

**Figure 11:**
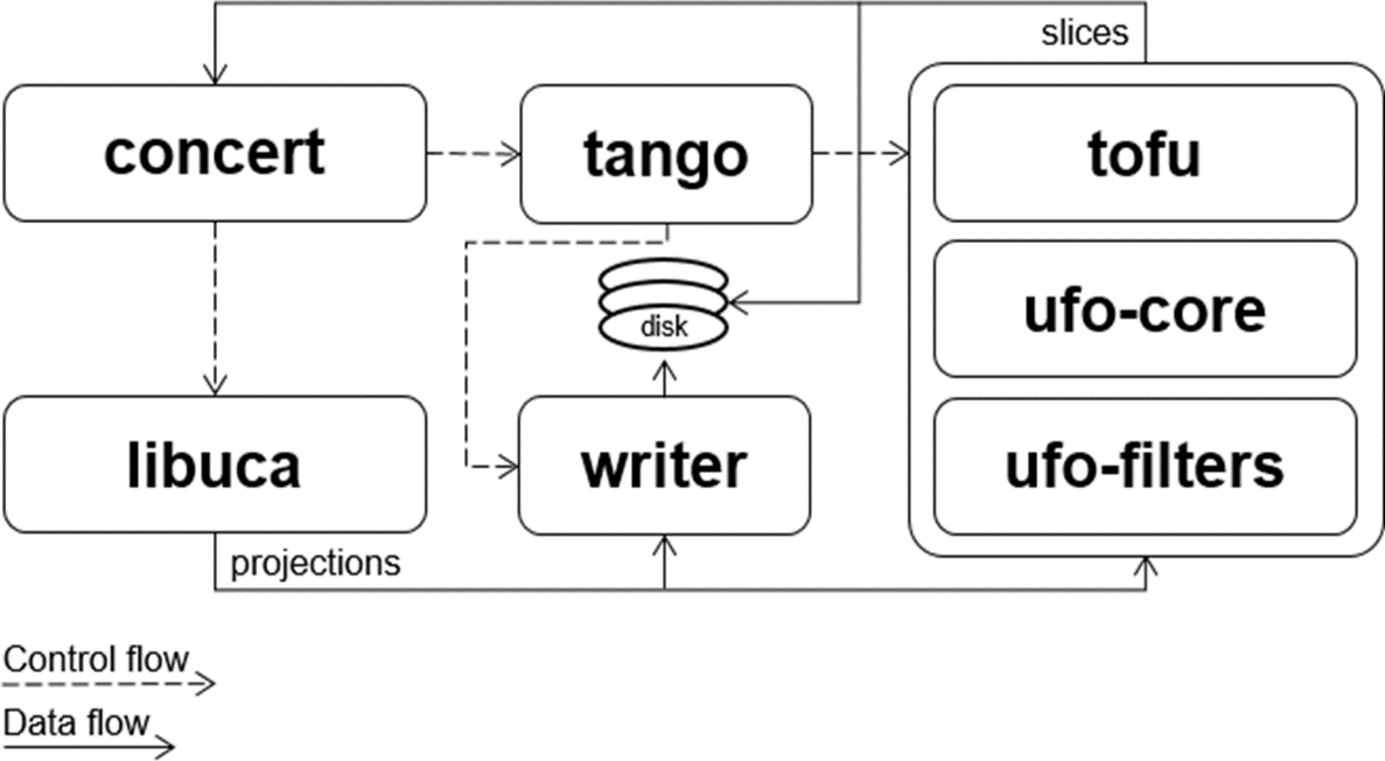
Diagram showing experiment control, data flow and how *Concert* uses different software packages. *Libuca* is used for accessing the cameras. *Tango* is used to control devices, image writing and also the online 3D reconstruction engine, which uses *tofu* to create the 3D reconstruction pipelines. They consist of OpenCL algorithm implementations in *ufo-filters* and are executed by *ufo-core*.

The beamline employs the Python-based control system *Concert* (Vogelgesang *et al.*, 2025 ▸) for high-level control of devices and experiments in a unified way. Low-level device-specific control is realized by *Tango* (Chaize *et al.*, 1999 ▸) to communicate with most of the devices on the beamline and *libuca* (Vogelgesang *et al.*, 2016 ▸; see also https://github.com/ufo-kit/libuca) to control cameras and acquire images. *Concert* can stream images to *tofu* (Faragó *et al.*, 2022 ▸), which creates online 3D reconstruction pipelines that run on GPUs thanks to the UFO framework consisting of the *ufo-core* and *ufo-filters* libraries (see Fig. 11 ▸).

To avoid errors, users enter values with physical quantities, device attributes have adjustable bounds and each device has an associated finite state machine to prevent unwanted sequences of actions. *Concert* includes routines for automatically setting up typical imaging experiments, such as focusing of camera optics and aligning the tomographic rotation axis. It also includes several experiment classes consisting of acquisitions (*e.g.* a tomography experiment consists of dark-field, flat-field and projection acquisitions), allowing the combination of different instruments with different data acquisition logic. Runtime changes to the acquisition procedures are possible thanks to the ability to dynamically add, swap and remove acquisitions, *e.g.* swap the order of dark-field and flat-field acquisitions, add a second set of flat-fields, *etc*. Experiments can have different add-ons responsible for, *e.g.* live preview, writing data to disk, and online 3D reconstruction. Add-ons can also be dynamically attached to and detached from experiments. These features make data acquisition very agile, requiring configuration rather than programming new experiment variations. Recently, *Concert* was upgraded to a decentralized version, which splits the workload between multiple computers and speeds up the overall data acquisition process. This is realized by establishing *ZeroMQ* (https://zeromq.org/) links between a dedicated camera server, a storage server and a high-performance 3D reconstruction server.

## 5. Detector pool

At IMAGE, mainly indirect detectors consisting of thin scintillator crystals coupled to visible light cameras via diffraction-limited optical microscopes are employed (Douissard *et al.*, 2012 ▸). Several types of scintillators are available, including LSO:Tb scintillators (on YbSO substrate) with thicknesses ranging from a few micrometres up to 24 µm (https://www.esrf.fr/files/live/sites/www/files/Industry/documentation/F2_Scintillators.pdf; Cecilia *et al.*, 2011 ▸) and LuAG:Ce (free standing) with thicknesses ranging between 50 µm and 200 µm (https://www.crytur.com/materials/luagce/). The scintillator thickness is chosen to match the depth of field of the microscope and the spatial resolution/effective pixel size of the camera. As far as the cameras are concerned, the beamline has several CMOS detectors available, differing in sensor size, pixel size and maximum frame rate (Table 5 ▸). When dealing with *operando* or *in situ* experiments, the monitoring of the process understudy is limited by the camera memory and by the dimensions of the FOV. In particular, the available memory limits the number of images that can be stored, which can be a significant challenge when high-speed imaging is required over long periods of time. The Phantom camera (288 GB memory) and the PCO.dimax (36 GB memory) can acquire up to 72 338 images (7.8 s) and 9000 images (4.9 s), respectively, to fill their memory in full frame mode.

Depending on the spatial resolution and frame rate required for the experiment, the optimal visible microscope can be selected from a range of options, detailed in Table 6 ▸, including the available objectives and achievable spatial resolutions. The latter has been calculated using the Rayleigh criterion, which defines the resolution limit as *R* = 1.22λ/NA, where λ is the wavelength emitted by the scintillator and NA is the numerical aperture of the microscope objective. All the microscopes available at IMAGE were manufactured from Optique Peter (https://optiquepeter.com/en/home/), while the macroscope was produced from Elya Solutions (https://elya.cz/en/).

For applications requiring large FOV and low spatial resolution, a Shad-o-box 1k HS detector (https://www.teledynedalsa.com/en/home/) is available, with an active surface of 5.7 cm × 6.4 cm (1152 pixel × 1300 pixel, pixel size of 49.5 µm) and a maximum frame rate of 30 frames s^−1^. Besides the indirect detectors, a pool of direct-converting large-area single-photon-counting detectors based on Medipix3 technology are available, namely Quad (2 × 2 array of Medipix3) and Lambda (6 × 2 array of Medipix3) detectors for X-rays (Pennicard *et al.*, 2011 ▸) where each individual Medipix3 chip is composed of 256 × 256 pixels (pixel size of 55 µm).

## 6. Exemplary experimental results

### 6.1. *Operando* tomography of vanadium redox flow batteries

Taking advantage of the high flux density available in filtered white beam mode, we have investigated the hydrogen evolution reaction (HER), which occurs in vanadium redox flow batteries (VRFB). These undesired reactions need intricate rebalancing of the electrolyte during cell operation (*e.g.* by using different additives), resulting in electrode degradation with a consequent lifetime reduction of the device and therefore leading to increased operational costs. Understanding the nature of nucleation points for HER is therefore crucial for developing a cell design that effectively mitigates these side reactions (Köble *et al.*, 2021 ▸; Eifert *et al.*, 2020 ▸).

**Figure 12:**
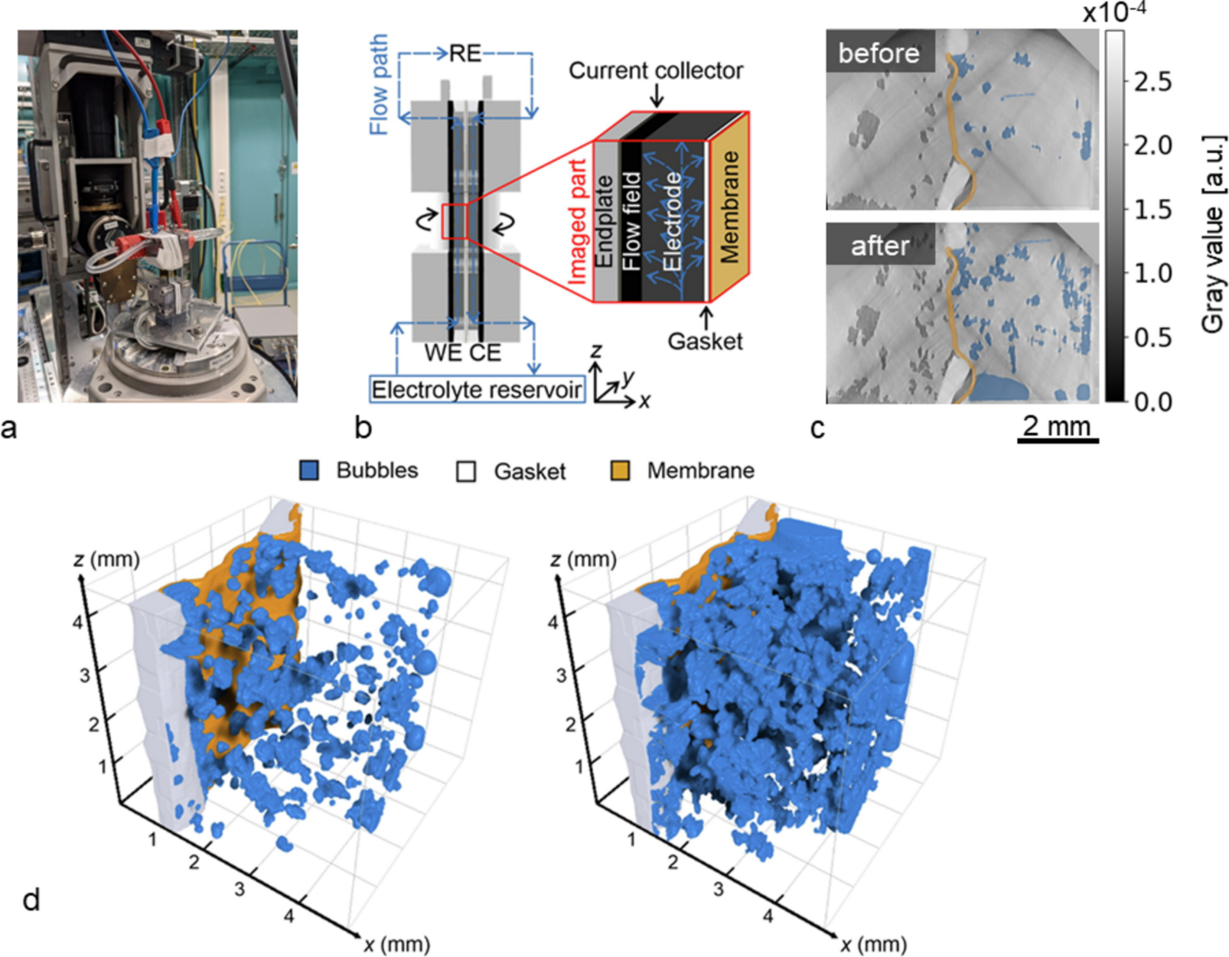
Hydrogen formation in a VRFB battery cell. (*a*) Cell positioned on the rotary stage, and (*b*) schematic details of the cell and the visualized part; (*c*) exemplary 2D slices of tomograms recorded before and after the electric potential is applied; (*d*) three-dimensional renderings of the segmentation results obtained from the tomograms recorded before and after a −300 mV potential is applied. The segmentation highlights the bubbles, the gasket and the membrane.

The tomography experiment was conducted in Experimental hutch 1, providing sufficient space for the power supply, electrolyte and cables of the cell, which with its height of 82 mm and a base of 35 mm × 30 mm was specifically designed to fit the tomography stage [Fig. 12 ▸(*a*)]. By using the filtered white beam at a magnetic wiggler field *B* = 1 T with 6 mm PG filters in combination with an indirect detector system featuring a LuAG:Ce 200 µm scintillating screen, a macroscope (with 3× magnification) and a Phantom v2640 camera, a frame rate of 200 frames s^−1^ over a FOV of 9.2 mm × 8.7 mm (H × V) was achieved. The study of the HER process was performed in ‘steady state’ steps by applying an increasing potential in steps of 25 mV for three minutes while no electrolyte flow was applied. After each potential step a local tomography scan [Fig. 12 ▸(*b*)] with 3000 projections was conducted to visualize the final state of the electrode after the HER period. Each tomography scan took 17 s, including motor movement. Exemplary slices through the reconstructed tomograms detailing the process are shown in Fig. 12 ▸(*c*), recorded before and after applying the final potential of −300 mV, respectively.

To quantify the distribution of hydrogen bubbles in 3D, we developed an AI-based segmentation approach. For this, multiple datasets were manually annotated to create a training set for a U-Net model (Ronneberger *et al.*, 2015 ▸) with a ResNet-34 backbone (He *et al.*, 2016 ▸). The trained model was then applied to effectively segment the bubbles. A 3D distribution of bubbles before and after the applied potential is shown in Fig. 12 ▸(*d*), as well as the individual components within the imaged volume (bubbles, membrane and gaskets). The comparison demonstrates a notable increase of the amount of bubbles following the HER phase, highlighting the obstruction caused by this side reaction in blocking significant portions of the electrode volume (Köble *et al.*, 2024 ▸; Schilling *et al.*, 2024 ▸).

### 6.2. High-throughput imaging of insects

Tomographic measurements of large numbers of small biological samples is a major application field of the IMAGE beamline. As an example of the image quality achievable during high-throughput tomography, we present the 3D tomography reconstruction of a grain weevil (*Sitophilus granarius*) preserved in 100% ethanol, scanned with pink beam. The measurements were performed at a 2.7 T magnetic field of the wiggler, yielding a maximum flux density at 16 keV with an energy bandwidth of 2%. The heat load on the DMM was reduced with 3 mm of pyrolytic graphite.

The tomography scan of the weevil was acquired with UFO-I at a 5× optical magnification and using the pco.dimax camera, resulting in an effective pixel size of 2.44 µm with a sample-to-detector distance of 90 mm. For the scan, 100 dark-field images, 100 flat-field images and 3000 equiangularly spaced radiographic projections were taken at 50 frames s^−1^ in an angular range of 180°, resulting in a measurement duration of 70 s.

**Figure 13:**
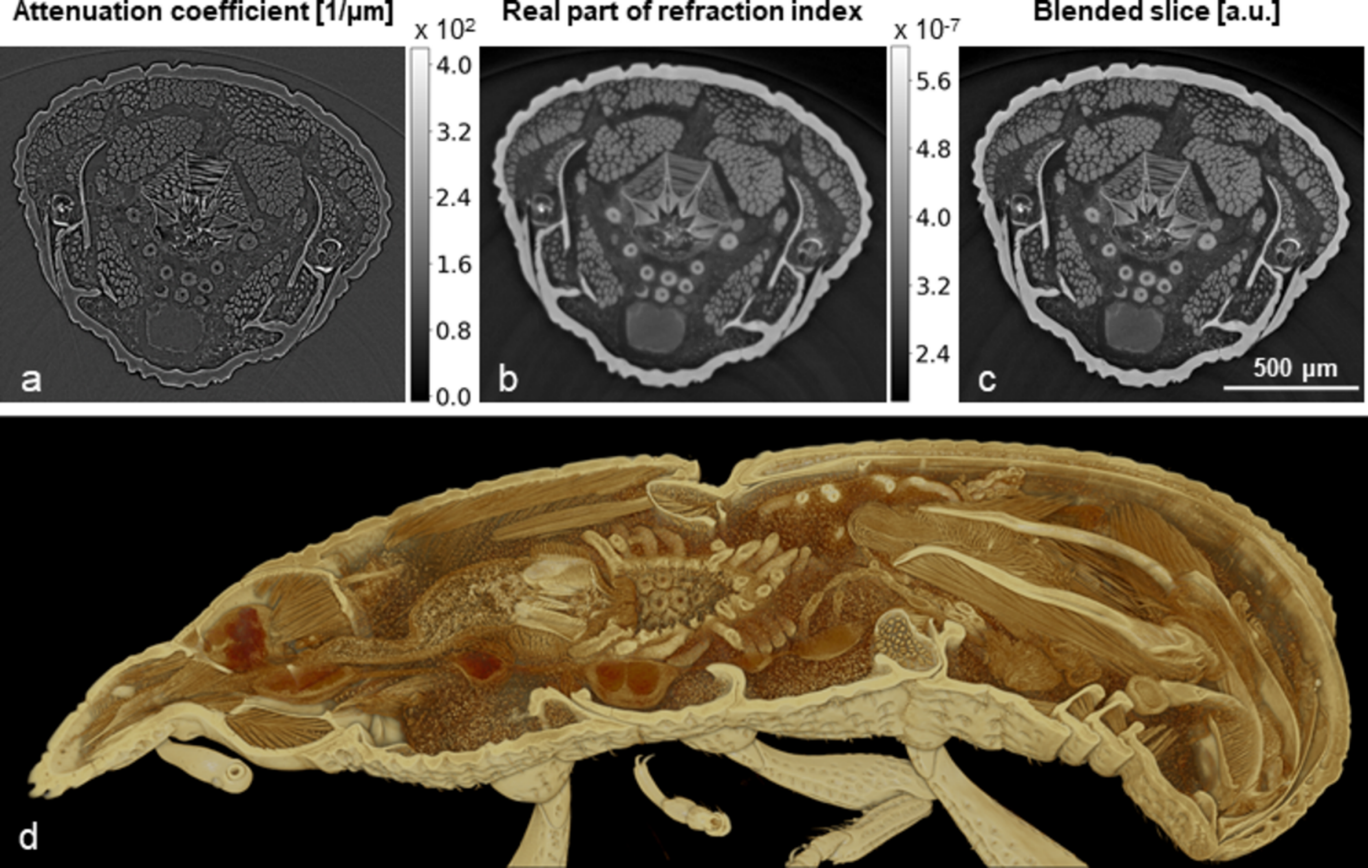
Fast tomography of a grain weevil (*Sitophilus granarius*). Slice of the tomogram after reconstruction of (*a*) attenuation coefficient with neglected phase effects, (*b*) real part of the refractive index, (*c*) blending of absorption and phase, and (*d*) cut 3D volume rendering. The grey scale for the real part of refraction index and the blended slice is the same.

The attenuation with neglected phase effects [Fig. 13 ▸(*a*)] together with the real part of the refractive index [Fig. 13 ▸(*b*)] were reconstructed by using the Paganin phase retrieval method (Paganin *et al.*, 2002 ▸). In addition, the blending of phase and absorption 3D reconstructions is included in Fig. 13 ▸(*c*). The complete image post-processing has been accomplished by using the functions available in *tofu* (Faragó *et al.*, 2024 ▸; Faragó *et al.*, 2022 ▸). Fig. 13 ▸(*d*) shows the 3D volume rendering of the blended tomogram by means of *Drishti 2.5.1* (Limaye, 2012 ▸).

The short scanning time combined with the high quality of the tomogram demonstrate IMAGE’s potential to acquire large numbers of datasets of insects and other millimetre-sized biological samples in short time. The voxel-wise weighted addition (blending) of the two reconstruction types provides a well balanced final 3D image contrast between both the higher absorbing specimen parts like the exoskeleton and the different soft tissues while still emphasizing the sharp boundaries between all body parts. To fuse the two images, we use a weighted average method, where we combine the absorption and phase images by using a weight factor (α) equal to 0.5 in the case of the arthropod. This makes the data particularly well suited for further post-processing like image segmentation with semi-automatic tools like *Biomedisa* (Lösel *et al.*, 2020 ▸) or AI-based fully automatic segmentation by dedicated neural networks (Lösel *et al.*, 2023 ▸; Jonsson, 2023 ▸; Toulkeridou *et al.*, 2023 ▸), which are important to process the hundreds and thousands of tomograms of high-throughput measurements.

Overall, IMAGE opens up excellent opportunities for large-scale systematic studies on the 3D morphology of biological samples, promising short scanning times and data that can be employed for studies on their biodiversity, evolutionary biology, development, functional morphology and biomimetics. Moreover, such data may be combined with other types of information, *e.g.* to investigate phenotype–genotype correlations or morphological changes due to environmental factors such as climate change. The first publications featuring high-throughput insect scans of the IMAGE beamline highlight cestode infected ants (Prebus *et al.*, 2023 ▸) defensive glands in termites (Thakur *et al.*, 2024 ▸) and the evolution of stinger shape in ants (Casadei-Ferreira *et al.*, 2024 ▸).

### 6.3. DCM application: Bragg magnifier conditioner

This section describes the test of a beam conditioner system based on Bragg magnifier (BM) optics, taking advantage of crystals cut with the asymmetry angle α close to the Bragg angle θ_B_ (Boettinger *et al.*, 1979 ▸; Spiecker *et al.*, 2023 ▸). When an X-ray beam impinges this set of lattice planes at an angle θ_B_ with the crystal surface at (θ_B_ − α), the diffracted beam profile is magnified by a factor *M* according to
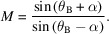
To reduce the heat load and thus any possible thermal stresses in the crystals giving rise to misalignments, the experiment was performed by using the beam monochromated by the DCM.

The BM crystal optics have the capability of enlarging X-ray beams to illuminate centimetre-large illuminated areas while maintaining the monochromaticity and coherence provided by the synchrotron beam. The application of BM conditioners is particularly interesting for fourth-generation synchrotron imaging beamlines, where typical beam sizes of only a few millimetres severely limit application to larger samples (Balewski *et al.*, 2004 ▸; Leemann & Eriksson, 2014 ▸).

The BM-based conditioner tested at IMAGE is composed of four single Si(220) crystals having an asymmetry angle of α = 5.92° and α = 0.94° for the first/third and second/fourth crystal, respectively, which altogether enlarge the beam profile in two dimensions in an in-line configuration. The device operates in the energy range 29–31 keV, where it provides a magnification factor between *M* = 34.5 and *M* = 198.5.

**Figure 14:**
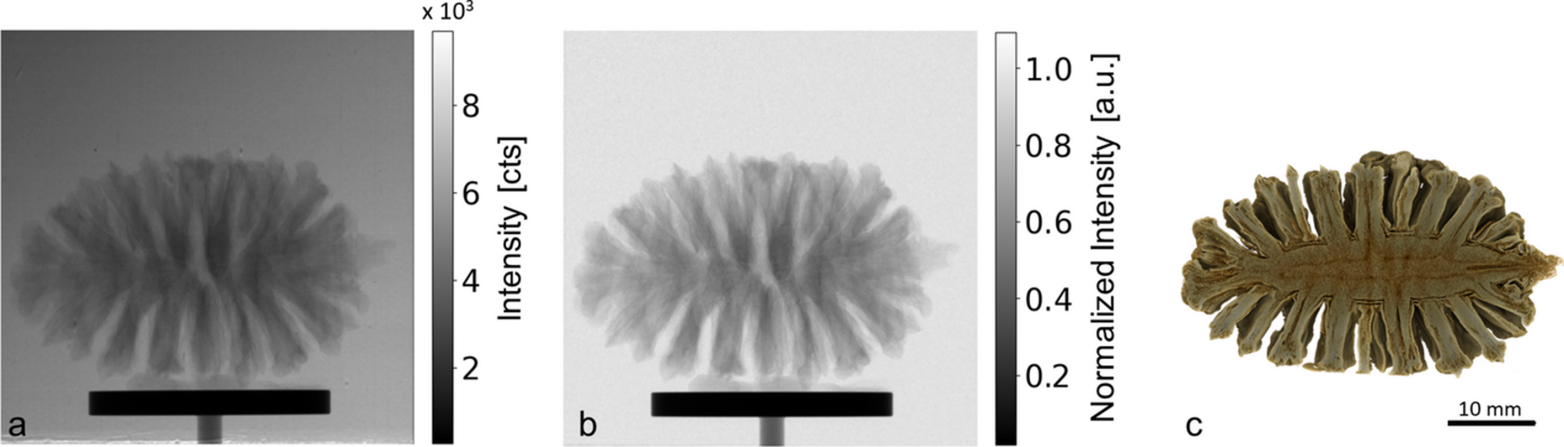
Tomographic measurement of a full pinecone with monochromated beam at 30.5 keV. The FOV of 5 cm × 5 cm (H × V) has been illuminated by the beam magnified by means of a Bragg-magnifier conditioner, achieving a flux corresponding to ∼500 X-ray photons pixel^−1^ s^−1^ (49.5 µm pixel size). The scale bar is equal in all of the images. (*a*) Projection image as recorded by the detector. (*b*) Flat-field-corrected image. (*c*) Cut in the centre volume rendering of the 3D tomography reconstruction obtained using *Drishti* (Limaye, 2012 ▸).

For the presented example, we used a photon energy of 30.5 keV leading to a magnification of *M* = 71.3. In this way, the magnified beam illuminated a large FOV (5 cm × 5 cm, H × V, pixel size 49.5 µm) of the Shad-o-box 1k HS detector (https://www.teledynedalsa.com/en/home/), the dimension of the magnified beam limited only by the size of the crystals used. As illustrated in Fig. 14 ▸, with the obtained large monochromated beam it was possible to acquire tomographic projection data of a full dried pine cone, recording 1000 projections with an integration time of 1 s per projection, resulting in a total measurement time of 17 min.

### 6.4. *In situ* lamino­graphy of Al alloy sheets during mechanical testing

The mechanical properties of sheet materials is of particular interest for many modern applications, *e.g.* transportation, but many relevant stress states cannot be observed in the typical rod-shaped samples suitable for conventional *in situ* computed tomography. Here, *in situ* lamino­graphy has proven unique capabilities for studying the relation of macroscopic deformation, stress and deformations fields, and microscopic features like the role of voids and secondary phase particles for crack initiation (Morgeneyer *et al.*, 2014 ▸; Kong *et al.*, 2022 ▸; Hurst *et al.*, 2023 ▸) in approximately two-dimensional samples with considerable lateral extension.

**Figure 15:**
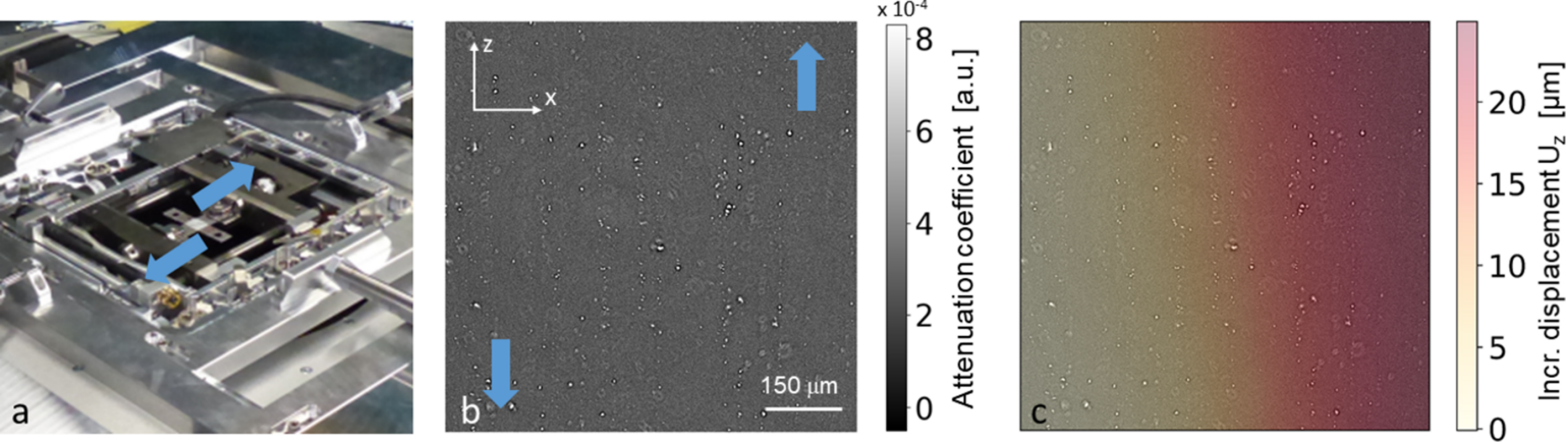
*In situ* lamino­graphy measurement for the characterization of deformation and microstructural change in a material under shear. The blue arrows indicate the loading direction. (*a*) Dedicated *in situ* loading device integrated into the LAMINO-II station. (*b*) Slice of the reconstructed sample volume showing the aluminium matrix (grey) and intermetallic particles (white) at the ROI at the sample centre. (*c*) Overlay of (*b*) with one component of the extracted displacement field.

Such an *in situ* lamino­graphy experiment and its results are as an example illustrated in Fig. 15 ▸. A dedicated loading device [see Fig. 15 ▸(*a*)] has been used in combination with LAMINO-II, enabling the acquisition of a series of lamino­graphic 3D measurements of a cross-shaped Al–Cu–Li alloy sample undergoing subsequent loading steps, due to the specially designed shape inducing a shear stress around the centre. The measurements were performed in pink beam mode (30 keV with 3 mm pyrolytic graphite filters), using a pco.edge5.5 camera (see Table 5 ▸). The 3600 radiographic projection images were acquired with a frame rate of 5 frames s^−1^, resulting in a total measurement time per load step of 13 min. The 3D reconstructions of the microstructure damage were performed with filtered back projection using the image processing toolkit *tofu* (Faragó *et al.*, 2022 ▸). As shown in Fig. 15 ▸(*b*), the reconstructed slices allow features such as particle cracking or cracks in the aluminium matrix to be identified. By means of digital image or digital volume correlation techniques, the deformation field induced by the loading can be extracted for the full course of the gradual loading of the material [Fig. 15 ▸(*c*)]. Here, the shown deformation field has been calculated using so-called projection digital image correlation (Kong *et al.*, 2022 ▸) using the image correlation software *SPAM* (Stamati *et al.*, 2021 ▸).

With its cable drag and acceptance of sufficiently large and heavy samples (in particular enabling the use of a tensile testing machine), the design of LAMINO-II and the infrastructure at IMAGE is well suited to perform such measurements in a systematic way, *i.e.* performing test series varying, *e.g.* material compositions, sample geometries, load paths *etc*., in a considerably automatized way and in reasonable time.

## 7. Conclusions

In this paper, we have presented the current state of the IMAGE beamline at the KIT Light Source, which focuses on applications in full-field hard X-ray imaging in materials and life sciences.

The combination of the provided high flux density and high energy photons with the available instrumentation installed in Experimental hutch 2 facilitates high-throughput tomography scans at the UFO-I/UFO-II station, optimized for processing thousands of samples per week. In addition, *in situ* and *operando* studies can be conducted at the LAMINO-II station, which offers unique possibilities for systematic studies, *e.g.in situ* tensile testing of sheet materials, as well as for hierarchical imaging of flat and laterally extended objects by screening large sample areas followed by zooming in at selected ROIs.

For more specialized experiments, which require, for example, the use of complementary instrumentation such as pumps for electrolyte solutions, furnaces, cryo-chambers or compression devices, Experimental hutch 1 provides the necessary space and a high degree of flexibility for customized set-ups.

IMAGE can provide X-ray photons with filtered white beam as well as monochromated (8–40 keV) by a DMM (d*E*/*E* ≃ 3%) or a DCM (d*E*/*E* ≃ 0.001%), which allows flexible beam conditions regarding energy resolution, heat load, speed and dose.

Altogether, the presented features make IMAGE a flexible tool for X-ray imaging, providing a pool of methods applicable to a wide range of scientific fields, where the current focus is in particular on materials science and life science. Nevertheless, the beamline is open to any interesting project, including environmental science, cultural heritage, archaeology and many more.

## Supplementary Material

Simulation of the DMM and DCM photon flux density (at 39 m from the source) for representative energies of 8 keV, 15 keV and 25 keV (see Fig. S1), to quantify the effect of the source divergence on the 2D photon flux density distribution. DOI: 10.1107/S1600577525003777/ye5068sup1.pdf

## Figures and Tables

**Table 1:** Main parameters of the CLIC superconducting wiggler, the insertion device at the IMAGE beamline

Period (mm)	51
Number of periods	34
Magnetic pole gap (mm)	18
On-axis magnetic field amplitude (T)	2.9
Source size (H × V) (mm)	1.254 × 0.065
Source divergence (H × V) (mrad)	1.374 × 0.123

**Table 2:** DM1, DM2 and DM3 components at IMAGE

Component	White beam mode	Pink beam mode	Monochromatic mode
Profile monitor sensor	Si photodiode with 10 µm W pinhole		
Sensor protection	200 µm aluminium	N.A.	N.A.
Fluorescence screen	500 µm CVD diamond	Yttrium oxide coated copper screen	
Intensity monitor	Quadrant Si photodiode		
Intensity monitor scatterer	500 µm CVD diamond		
Intensity monitor foil sensor	500 µm pyrolytic graphite	250 µm glassy carbon	12.5 µm aluminium

**Table 3:** List of diagnostic elements installed at DM1, DM2 and DM3

Module	DM1	DM2	DM3
Profile monitor		X	
Intensity monitor foil		X	X
Intensity monitor scatterer	X		
Fluorescence screen	X	X	X

**Table 4:** Main characteristics of UFO-I and UFO-II stations

	UFO-I	UFO-II
Weight/area	950 kg/1050 mm × 2150 mm	5200 kg/1500 mm × 2500 mm
Rotary stage maximum	RT150AS (https://www.labmotionsystems.com/)	RT150AS (https://www.labmotionsystems.com/)
Speed	200 rev min^−1^	200 rev min^−1^
Sphere of confusion	<150 nm	<150 nm
Maximum load	43 kg	43 kg
Camera	pco.dimax	pco.edge 5.5 CLHS and Phantom v2640
Microscope	Double branch microscope	Fivefold branch microscope
Stored samples	Up to 49	Up to 2200

**Table 5:** CMOS cameras available at IMAGE

Camera	Maximum speed at full frame (frame s^−1^)	Number of pixels (H × V)	Pixel size (µm)
pco.edge 5.5 (https://www.excelitas.com/de/product-category/pco)	50 (global shutter)	2560 × 2160	6.5 × 6.5
pco.edge 5.5 camera link high speed (CLHS)	100 (rolling shutter, global shutter)	2560 × 2160	6.5 × 6.5
pco.dimax HS4	2277	2000 × 2000	11 × 11
AMETEK Phantom v2640 (https://www.phantomhighspeed.com/)	6600	2048 × 1944	13.5 × 13.5

**Table 6:** Configurations of microscopes for X-ray indirect detector systems available at IMAGE

Device†	Objective	NA / resolution limit
Monochromatic microscope	Nikon Uplsapo 4×	0.16 / 2.1 µm
	Nikon Uplsapo 10×	0.40 / 0.84 µm
	Nikon Uplsapo 20×	0.75 / 0.45 µm

Double branch, White beam microscope	Mitutoyo 2×	0.055 / 6.0 µm
	Mitutoyo 5×	0.14 / 2.4 µm
	Mitutoyo 10×	0.28 / 1.2 µm

White beam macroscope	Nikon Nikkor 85 mm f / 1.4D (*M* = 3×)	0.36 / 0.84 µm

LAMINO-II microscope, White beam microscope	Mitutoyo 2×	0.055 / 6.0 µm
	Mitutoyo 5×	0.14 / 2.4 µm
	Mitutoyo 7.5×	0.21 / 1.6 µm
	Mitutoyo 10×	0.28 / 1.2 µm
	Mitutoyo 20×	0.42 / 0.8 µm

Fivefold branch, White beam microscope	Mitutoyo 1×	0.035 / 9.6 µm
	Mitutoyo 2×	0.055 / 6.0 µm
	Mitutoyo 5× HR	0.21 / 1.6 µm
	Mitutoyo 10×	0.28 / 1.2 µm
	Mitutoyo 20×	0.42 / 0.8 µm

† All the microscopes mount tube lenses that contribute to the total magnification, depending on their distance from the objective. The monochromatic and the LAMINO-II microscopes mount a 1× tube lens. The fivefold microscope has a 1× and a 1.5× tube lens, for the high spatial resolution and for the low spatial resolution branch, respectively. The double branch microscope has a tube lens that provides a 0.9× magnification. The microscopes are generally used without eyepiece. However, they can offer the possibility to mount 2× and 2.5× eyepieces, respectively.

## Footnotes

1 The acronym UFO stands for Ultra Fast X-ray imaging of scientific processes with On-line assessment and data-driven process control.

2 The injection chamber, the Navitar long-distance microscope objective and the Photron Fastcam SA-Z are not part of the instrumentation available at IMAGE. They belong to FAU University of Erlangen-Nuremberg.
