# Supplementary material for: The IMAGE beamline at the KIT Light Source
Source: J Synchrotron Radiat. 2025 Jun 2;32(Pt 4):1036–51. doi: 10.1107/S1600577525003777 (PMC12236244; doi:10.1107/S1600577525003777)
Supplement: Supplementary file 1 [file s-32-01036-sup1.pdf]

JOURNAL OF SYNCHROTRON RADIATION

Volume 32 (2025)

Supporting information for article:

The IMAGE beamline at the KIT Light Source

Angelica Cecilia, Rolf Simon, Elias Hamann, Marcus Zuber, Tomas Farago, Daniel Haenschke, Mathias Hurst, Thomas van de Kamp, Sondes Bauer, Rebecca Spiecker, Mateusz Czyzycki, Sergei Gasilov, Alexey Ershov, Jan-Thorsten Reszat and Tilo Baumbach

Here we provide the simulation of the DMM and DCM photon flux density (at 39 m from the source) for representative energies of 8 keV, 15 keV and 25 keV (see Fig. S1), to quantify the effect of the source divergence on the 2D photon flux density distribution.

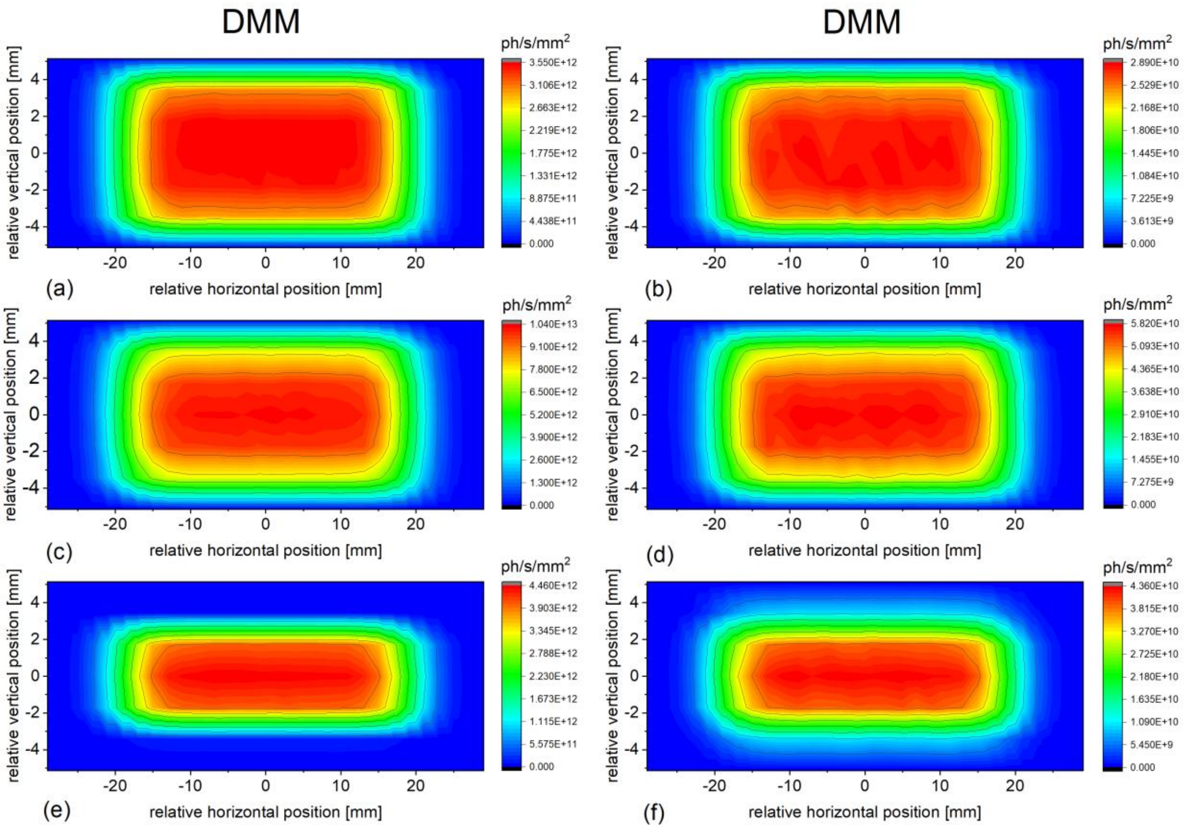

Figure S1 Photon flux density calculated at 39 m for the DMM at (a) 8 keV, (c) 15 keV and (e) 25 keV and for the DCM at (b) 8 keV, (d) 15 keV and (f) 25 keV. Calculations performed with XTRACE (Bauer et al., 2007).
